# Assessing respiratory viral exclusion and affinity interactions through co-infection incidence in a pediatric population during the 2022 resurgence of influenza and RSV

**DOI:** 10.3389/fcimb.2023.1208235

**Published:** 2023-06-14

**Authors:** Maxwell D. Weidmann, Daniel A. Green, Gregory J. Berry, Fann Wu

**Affiliations:** Department of Pathology & Cell Biology, Columbia University Irving Medical Center, New York, NY, United States

**Keywords:** COVID-19, SARS-CoV-2, multiplex, co-infection, respiratory, virus, influenza, RSV

## Abstract

**Introduction:**

In the Northeast US, respiratory viruses such as influenza and respiratory syncytial virus (RSV), which were largely suppressed by COVID-19-related social distancing, made an unprecedented resurgence during 2022, leading to a substantial rise in viral co-infections. However, the relative rates of co-infection with seasonal respiratory viruses over this period have not been assessed.

**Methods:**

Here we reviewed multiplex respiratory viral PCR data (BioFire FilmArray™ Respiratory Panel v2.1 [RPP]) from patients with respiratory symptoms presenting to our medical center in New York City to assess co-infection rates of respiratory viruses, which were baselined to total rates of infection for each virus. We examined trends in monthly RPP data from adults and children during November 2021 through December 2022 to capture the full seasonal dynamics of respiratory viruses across periods of low and high prevalence.

**Results:**

Of 50,022 RPPs performed for 34,610 patients, 44% were positive for at least one target, and 67% of these were from children. The overwhelming majority of co-infections (93%) were seen among children, for whom 21% of positive RPPs had two or more viruses detected, as compared to just 4% in adults. Relative to children for whom RPPs were ordered, children with co-infections were younger (3.0 vs 4.5 years) and more likely to be seen in the ED or outpatient settings than inpatient and ICU settings. In children, most viral co-infections were found at significantly reduced rates relative to that expected from the incidence of each virus, especially those involving SARS-CoV-2 and influenza. SARS-CoV-2 positive children had an 85%, 65% and 58% reduced rate of co-infection with influenza, RSV, and Rhino/enteroviruses, respectively, after compensating for the incidence of infection with each virus (p< 0.001).

**Discussion:**

Our results demonstrate that most respiratory viruses peaked in different months and present in co-infections less than would be expected based on overall rates of infection, suggesting a viral exclusionary effect between most seasonal respiratory viruses, including SARS-CoV-2, influenza and RSV. We also demonstrate the significant burden of respiratory viral co-infections among children. Further work is necessary to understand what predisposes certain patients for viral co-infection despite this exclusionary effect.

## 1 Introduction

Since the start of COVID-19 pandemic 3 years ago, there have been over 750 million confirmed cases and estimates of 44% of the global population having been infected by the end of 2021. This has transformed global awareness of respiratory viral illness (Organization, G.W.H, 2020; Collaborators, 2022). Amidst a surge of research on SARS-CoV-2, there have also been a wealth of studies assessing the effect of co-infections, or secondary infections, in patients with COVID-19 (Alhumaid et al., 2021; Dao et al., 2021; Kim et al., 2021; Kinoshita et al., 2021; Sreenath et al., 2021; Alhumaid et al., 2022; Hedberg et al., 2022; Krumbein et al., 2023). For the first year of the pandemic, lockdown measures appeared to lower the incidence of most seasonal respiratory viruses (Uhteg et al., 2022), but gradual relaxation of these measures and social distancing norms have led to their re-emergence.

In this setting, assays that can detect multiple respiratory viral and/or bacterial co-infections can play an important role in both treatment decisions and infection control measures. Multiplex molecular assays, such as the FilmArray™ Respiratory Panel v2.1 (RPP), have become increasingly popular due to their ability to rapidly assess for up to twenty pathogen-specific targets simultaneously (Andersson et al., 2014; Hanson and Couturier, 2016). There has been some controversy around the clinical utility of such panels for regular use in the outpatient pediatric setting, amidst concerns for diagnostic stewardship, prior to the COVID-19 pandemic (Esposito et al., 2019; Hanson et al., 2020). More recently, there has been a shift in the role of multiplex polymerase-chain reaction (PCR) assays in rapidly differentiating cases of SARS-CoV-2 infection from other respiratory viruses for public health purposes, and to identify common and treatable viral etiologies such as influenza. However, the role of multiplex respiratory viral testing to assess viral co-infection, particularly the interaction of SARS-CoV-2 with other respiratory viruses, has received relatively little attention.

Multiplex PCR assays also represent a novel opportunity to study the interaction of viruses in real time as they move through human populations. Of all viral co-infections involving SARS-CoV-2, influenza has received the most attention, with a meta-analysis from early in the pandemic demonstrating an overall co-infection rate of 0.7%, but much higher rates in children (3.2%) (Dao et al., 2021). Yet children displayed a very different pattern of respiratory co-infection prevalence with a more recent metanalysis demonstrating the highest prevalence from RSV (1.7%) and rhinovirus (1.0%), with influenza as third most prevalent at only 0.5% of overall SARS-CoV-2 infections (Alhumaid et al., 2022). In contrast, another metanalysis looking at viral co-infections in all age groups found influenza overall ranking third amongst viral co-infections with SARS-CoV-2 (1.2% prevalence), with EBV (1.8%) and HHV6 (1.6%) being more common (Alhumaid et al., 2021). Several factors contribute to the diversity in the rates and types of viral respiratory co-infection seen, including the background rates of infection with each virus, mechanisms of viral exclusion or predisposition for co-infection. The advent of multiplex viral panels allows for relative ease in assessing the background rates of mono vs. co-infection for a given patient population, yet few studies have formally attempted this.

By normalizing for the probability of mono-infection, when comparing the relative incidence of various forms of viral co-infection, we can therefore gain novel insights into viral interactions within human hosts that have thus far only been studied in animal models. Horemheb-Rubio et al. (2022) conducted such an examination of respiratory viral interactions from 2010-2019 in Europe found relatively few synergistic viral interactions, such as between influenza H3N2 and parainfluenza virus 4 or HCoV-NL63 and parainfluenza virus 1, and predominantly viral exclusion between influenza and RSV, rhinovirus and most parainfluenza viruses (Horemheb-Rubio et al., 2022). However, such an analysis has not been conducted on a pediatric population, which have been shown to have higher rates of respiratory viral co-infection (Dao et al., 2021; Chen and Er, 2022; Krumbein et al., 2023), in general, nor been conducted since the beginning of the COVID-19 pandemic and therefore systematically assessed such viral co-infection dynamics in patients with SARS-CoV-2. The resurgence of respiratory viruses seen over the past two Northern Hemisphere winter seasons represents a unique opportunity to study their co-infection rates normalized to background mono-infection.

## 2 Methods

### 2.1 Study population

We performed retrospective analysis of a total of 50,022 BioFire FilmArray™ Respiratory Panel v2.1 tests (noted as Respiratory Pathogen Panel or RPP) (BioFire^®^ Diagnostics, Salt Lake City, UT, USA) were performed over November 1^st^, 2021 through December 31^st^, 2022 for 34,610 patients seen at one of several sites at Columbia University Irving Medical Center. Our hospital has instituted policies wherein pediatric patients with upper respiratory symptoms who are seen in the ED and are planned for admission, or inpatients who develop respiratory symptoms, are screened with the BioFire Respiratory Pathogen Panel 2.1 [RPP]. Duplicate results were excluded from the analysis. For patients with multiple positive RPPs, each positive RPP was considered as a separate episode of infection, in order to include new targets detected throughout a patient’s hospital course or for different encounters. All RPP tests were performed using nasopharyngeal (NP) swabs on patients suspected of respiratory tract infection. Subsequent analysis was performed solely on the pediatric population, defined as age less than 18 years at time of NP swab collection.

### 2.2 BioFire FilmArray™ Respiratory Panel v2.1

Nasopharyngeal swab samples were collected in viral transport media and analyzed with the BioFire FilmArray™ Respiratory Panel v2.1 as per the manufacturer’s instructions, which includes nucleic acid extraction, non-specific amplification, target-specific amplification, target detection and automatic interpretation of each target as detected, not detected or invalid from melting curve data by BioFire FilmArray™ software. The Panel consists of 21 targets, four of which are specific to bacteria (*Bordetella pertussis*, *Bordetella parapertussis*, *Chlamydia pneumoniae* and *Mycoplasma pneumoniae*) and the remaining specific for viruses, including SARS-CoV-2, influenza viruses (A, B, A H3, A H1 2009 variant), RSV, parainfluenza viruses (types 1-4), human rhinovirus/enterovirus, human metapneumovirus (HMpv), and non-SARS coronaviruses (229E, NL63, OC43, and HKU1).

### 2.3 Data analysis

Data were imported from Cerner (Kansas City, MO) using Discern Analytics 2.0 software. Raw data were analyzed using Microsoft Excel. R Studio (Posit Software, PBC) was used for additional statistical analyses including Chi-squared testing to assess for significance between categorical variables, and Pearson correlation coefficients were used to examine the linear correlation between monthly incidence of viral co-infections. Viral predisposition or exclusion of co-infection with another virus was assessed by comparing the probability of co-infection involving viruses X and Y with the probability of random co-incidence of each viral infection in the same individual as described in (Horemheb-Rubio et al., 2022). While these authors calculated their “co-infection exclusion score” initially for each month of data ((Horemheb-Rubio et al., 2022), supplemental 1.1), and then compiled monthly values into one composite score, here we calculated a similar ratio from our full dataset. If this Viral Co-infection Ratio (VCR) value equals 1, the chance of co-infection is the same as expected from the incidence of each virus, values less than 1 represent a reduced chance of co-infection relative to expected and values greater than one represent an increase relative to expected. We calculated the percent change in probability relative to expected for a particular viral co-infection pair *via* the equation:


(−1 + VCR)*100

## 3 Results

### 3.1 Increased rates of RPP positivity and co-infection in pediatric patients

While children (<18yrs) represented a minority of patients tested by RPP (45.3%, **Table 1**), and a minority of RPPs tested overall were in children (47.0%), they represented a significantly higher proportion of patients with positive RPP (at least one target positive) (69.6%, p<0.001) as well as positive RPPs overall (71.8%, p< 0.001; **Table 1**). RPP ordering rates were similar in pediatric patients relative to adults (1.5 vs. 1.4 RPPs/patient), indicating that repeat RPP ordering for a single patient was not frequent both amongst children and adults, however pediatric patients had a three-fold higher rate of positive RPPs per patient (15,790/15,675 = 1.01) relative to adults (6,211/18,935 = 0.33) (p< 0.001).

**Table 1:** RPP ordering, positivity and co-infections.

Patients/Testing	Total	Pediatric (%)	Adult (%)
**Patients**	34,610	15,675 (45.3)	18,935 (54.7)
**Patients Positive**	18,207	12,681 (69.6)	5,526 (30.4)
**Patients Positivity Rate**	52.6%	80.9%*	29.2%*
**RPP Total**	50,022	23,529 (47.0)	26,495 (53.0)
**RPP Positive**	22,001	15,790 (71.8)	6,211 (28.2)
**RPP Positivity Rate**	44.0%	67.1%*	23.4%*
**Mono-infection**	18,496	12,527 (67.7)**	5,969 (32.3)**
**Co-infection**	3,505	3,263 (93.1)**	242 (6.9)**
**Double**	3,169	2,941 (92.8)**	228 (7.2)**
**Triple**	317	303 (95.6)**	14 (4.4)**
**Quadruple**	17	17 (100)	0 (0)
**Quintuple**	2	2 (100)	0 (0)

Patients with an RPP ordered, those with positive RPPs, number of RPPs ordered and positive, as well as the number of RPPs positive for a single (mono-infection) or more than one target (Co-infection) are listed for overall and pediatric patient populations. Percent of each category composed of pediatric patients (<18 years) are listed in the rightmost column. Positivity rates are listed as percentages of patients with positive RPPs or positive RPPs out of the total number in each category. *p< 0.001 for pediatric vs. adult, **p< 0.001 for proportion of each category to total RPP for pediatric compared to adult.

**Figure 1:**
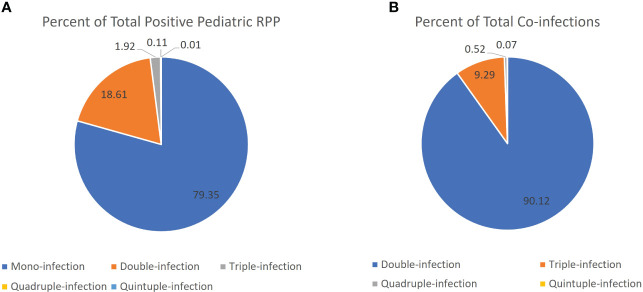
Proportion of positive RPPs that were positive for a single vs. multiple viral targets. **(A)** Pie chart comparing percentage of mono-infection (single target positive) vs. co-infection (two or more targets positive) out of total positive RPP in the pediatric patient population. **(B)** Pie chart comparing the percentage of RPPs positive with two or more targets positive out of the total number of RPPs with multiple targets (presumed co-infections) in the pediatric patient population.

The overall RPP positivity rate was 2.7-fold higher in children relative to adults (67.1% vs. 23.4%, p< 0.001). Further, the percentage of positive RPPs with multiple targets detected (co-infections) in pediatric patients (20.7%, 3,263/15790) was 5.3-fold higher relative to adults (3.9%, 242/6211) (p< 0.001), with significantly higher proportions of two, three and four target-positive RPPs in pediatric patients (**Figure 1B**). Most positive RPPs contained a single target positive, both for overall and pediatric patients (**Table 1**; **Figure 1A**).

### 3.2 Demographic characteristics of pediatric patients with RPP

The majority of pediatric patients with RPPs ordered were younger than 5 years of age (mean 4.5 years ± 0.06 95% CI), with a trend of lower age for those with positive RPP (3.9 years ± 0.06 95% CI), and particularly those with multiple targets positive (mean 3.0 years ± 0.10 95% CI; **Table 2**). Pediatric patients with RPPs ordered, as well as positive RPPs, were significantly more likely to be male (p< 0.001 and p< 0.001), for whom there was also a trend towards a higher rate of co-infection (**Table 2**). The majority of RPPs for pediatric patients were ordered from the ED (65.0%; **Table 2**), with significantly higher proportions of positive results and co-infection among ED patients (p< 0.001), with corresponding decreases in positive RPPs and co-infections from inpatient units. When stratifying by location, positivity rates and co-infections were highest among outpatient clinics (93.5% and 20.8%, respectively).

**Table 2:** Positive Respiratory Pathogen Panel demographics and level of care in pediatric patients.

	Demographics			Location of RPP				
	**Mean Age**	**Male (%)**	**Total Pts**	**ED (%)**	**Inpatient (%)**	**Outpatient (%)**​	**ICU (%)**	**RPPs**
**RPP Pediatric**	4.5	8599 (**54.2**)	15675	15297 (**65.0**)	6858 (**29.1**)	985 (**5.8**)	389 (**2.4**)	23529​
**RPP Positive Pediatric**​	3.9	7006 (**55.2**)	12681	11353 (**71.9**)	3516 (**22.3**)	921 (**5.8**)	142 (**1.9**)	15790​
**Co-infections Pediatric**​	3.0	1606 (**54.5**)	2948	2390 (**73.3**)	668 (**20.5**)	205 (**6.3**)	27 (**0.8**)	3263
		**RPP Positivity Rate (%)**​		74.2	51.2	93.5	36.5	
		**RPP Co-infection Rate (%)**		15.6	9.7	20.8	6.9	

Demographics are reported respective to the age, gender and total number of pediatric patients with RPP ordered, positive or multiple targets positive (presumed co-infection). Location of RPP ordering is reported as number of RPPs ordered total, the number of positive RPPs, and the number of RPPs with multiple targets positive at each location. Percentages of the total RPPs in each category are also reported in parentheses for each category.

### 3.3 Distinct trends in respiratory viral infections over time in pediatric patients

**Figure 2:**
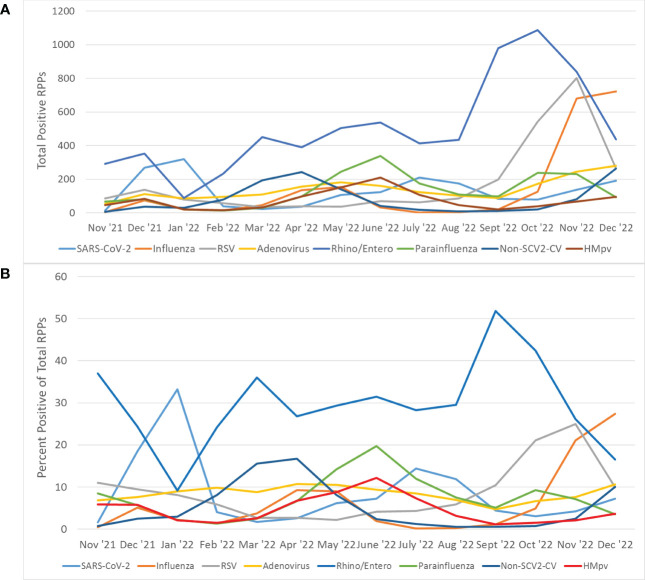
Trends overall viral infections in patients with RPP. **(A)** The number of total positive RPPs from each viral category are reported for each month of the study period. **(B)** The percentage of total RPPs ordered each month that were positive for each viral category was plotted for each month of the study period. Rhino/enterovirus was excluded for purposes of visualization. The influenza category refers to RPPs positive for any of the influenza A (non-subtyped), influenza A H3, influenza A H1 2009, and influenza B targets. The parainfluenza category refers to RPPs positive for any of the parainfluenza viruses 1-4. The Non-SCV-2 category refers to RPPs positive for any of the targets specific for HKU1, NL63, OC43 or 229E Coronaviruses.

Assessing monthly trends, a wide range of positivity rates among pediatric patients was observed, following distinct seasonal patterns for most viruses (**Figure 2**). Rhinovirus/enterovirus demonstrated the highest incidence during all but two months of the study period and peaked in September 2022 (**Figure 2A**). SARS-CoV-2 demonstrated peaks in January and July-August 2022, whereas seasonal coronaviruses peaked in March-April 2022. Influenza viruses peaked in April-May and December 2022, while metapneumovirus (HMpv) and parainfluenza viruses peaked in June 2022, and RSV peaked in November 2022.

Pairwise Pearson correlations between each of the viral categories depicted in **Figure 2** (**Table 3**) were assessed. Of 28 pairwise correlations, 17 were negative (inverse) and 11 were positive. SARS-CoV-2 showed a strong negative correlation (defined as<-0.5) with Rhino/enterovirus and a moderately negative correlation with seasonal coronaviruses (between -0.3 and -0.5). Influenza showed moderate positive correlations with RSV, adenovirus and seasonal coronaviruses, but a moderate negative correlation with rhinovirus/enterovirus. There was also a strong negative correlation between adenovirus and rhinovirus/enterovirus, as well as strong positive correlations between adenovirus and seasonal coronaviruses, as well as HMpv and parainfluenza viruses (**Table 3**).

**Table 3:** Pearson Correlation in viral incidence trends over study period.

	SARS-CoV-2	Influenza	RSV	Adenovirus	Rhino/Entero	Parainfluenza	Non-SCV2-CV
Influenza	-0.18						
RSV	-0.12	**0.39**					
Adenovirus	0.05	**0.39**	**-0.49**				
Rhino/Enterovirus	-0.64	**-0.36**	0.16	-0.67			
Parainfluenza	-0.15	-0.15	-0.13	0.06	0.24		
Non-SCV-CV	**-0.31**	**0.33**	**-0.46**	0.70	-0.22	**-0.31**	
HMpv	-0.03	-0.11	**-0.49**	**0.41**	-0.08	0.83	0.04

The incidence rates of each viral category, by month, over the study period (as depicted in **Figure 1B**) were compared by Pearson correlation, with coefficients reported for each pair. Correlations of moderate strength (0.30-0.50) are bolded in green (positive correlation) and red (negative correlation). Strong correlations (>0.50) are highlighted in either green (positive) or red (negative).​

### 3.4 SARS-CoV-2 demonstrates viral exclusion and low rates of respiratory viral co-infection

**Figure 3:**
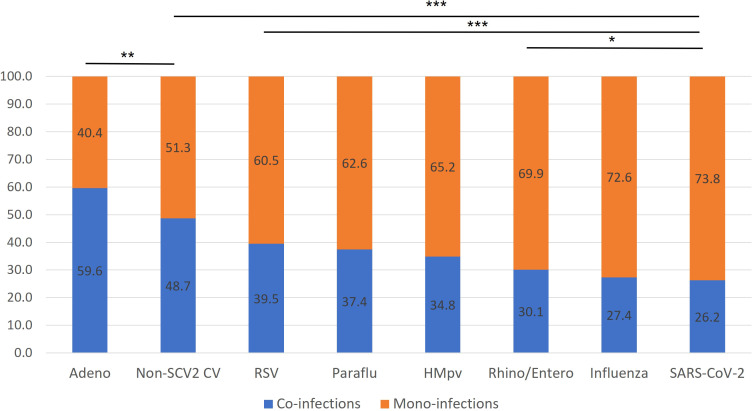
Rates of mono-infection vs. co-infection by virus. The percentage of total RPPs positive for each viral category, positive for only a single target (presumed mono-infection) or multiple targets (presumed co-infection) are listed for each viral category. p-values correspond to airs of specific viral categories. *p< 0.05, **p< 0.01, ***p< 0.001.

We found a wide variety of co-infection relative to overall infection percentages for individual viral categories, ranging from 59.6% (adenovirus) to 26.2% (SARS-CoV-2, **Figure 3**). SARS-CoV-2 showed a significantly lower co-infection proportion than every other viral category, except it did not have a significantly lower co-infection rate than influenza, which was next lowest at 27.4%. Adenovirus had a significantly higher co-infection proportion than every other viral category, including seasonal coronaviruses, which had the next highest co-infection proportion at 48.7% (p< 0.01).

**Figure 4:**
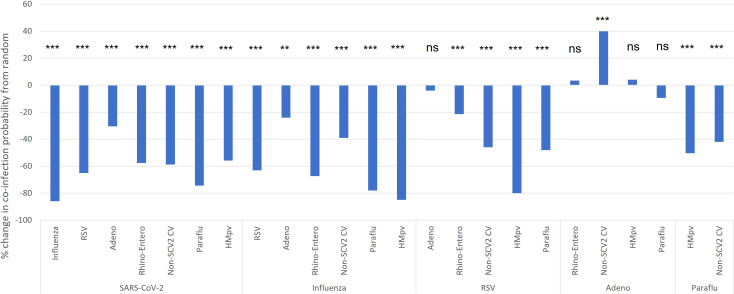
Pediatric co-infection probability relative to expected based on overall infection rate. The probability of viral co-infection was calculated based on the overall incidence of each co-infection pair, and compared to the expected probability of each co-infection type based on the incidence of each virus category as would be expected from stochastic interactions (random chance). *p< 0.05, **p< 0.01, ***p< 0.001. ns, not significant.

These findings aligned closely with our comparison of the probability of specific viral co-infections, relative to the probability of co-infection based on the random interaction of each virus involved, based on their overall prevalence in the study population (**Figure 4**). SARS-CoV-2, which had the lowest co-infection proportion (**Figure 3**), was found to have a significantly decreased probability of co-infection with every other type of respiratory virus assessed. Amongst co-infections with SARS-CoV-2, the greatest effect was seen with influenza, where there was an 86% lower than expected probability of co-infection, and the least effect with adenovirus, with a 30% reduction from expected (**Figure 4**). Influenza also demonstrated a significantly lower probability than expected of co-infection with any other virus assessed, from the highest probability with adenovirus, as a 21% reduction from expected, to the lowest with SARS-CoV-2. RSV demonstrated viral exclusion with every other category except adenovirus, with no significant difference from expected, and the largest reduction in probability was seen with HMpv at -79.9%. In general, most viral pairs assessed showed lower-than-expected probability of co-infection, except for adenovirus with seasonal coronaviruses, with a 39.9% increase from expected, while there was no significant difference from the expected co-infection probability for adenovirus co-infection with RSV, rhinovirus/enterovirus, and HMpv (**Figure 4**). Of note, we did not assess several viral pairs for whom there was no significant Pearson correlation coefficient seen (**Table 2**)

## 4 Discussion

This study is the first since the onset of the COVID-19 pandemic to assess respiratory viral co-infections while controlling for viral incidence in pediatric patients. Interestingly, our findings indicate that most common respiratory viruses are found in co-infection much more rarely than would expected based on their overall incidence. This finding is consistent with the assessment of incidence rates of individual RPP viral categories on a monthly basis, which demonstrated distinct peaks in incidence for each viral category, except for parainfluenza and HMpv. SARS-CoV-2, which has never been assessed by this method of estimating viral interactions in human subjects, was found to have a particularly high tendency for viral exclusion in all the viral categories assessed here. Influenza and RSV also demonstrated viral exclusion or no interaction for all viruses tested.

Influenza and SARS-CoV-2 co-infection in the hospitalized US pediatric population showed similarly low rates during the 2021-2022 influenza season as we saw for our overall pediatric patient population who received RPP, with 5.6% and 5.4% of influenza patients demonstrating SARS-CoV-2 co-infection, respectively (Adams et al., 2022). Despite this low co-infection rate, national data have demonstrated that co-infection carries a far higher risk of mortality, with the mortality rate of co-infected pediatric inpatients nearly three-fold higher (21.9%) than those infected with influenza alone (7.7%) (Adams et al., 2022). While there was no significant difference in rates of anti-viral therapy for patients hospitalized with co-infection vs. influenza alone, only one of the seven (14%) children who died with SARS-CoV-2 and influenza co-infection had received antiviral therapy for influenza, relative to 46% in the influenza-only population (Adams et al., 2022). Early administration of anti-influenza therapy may therefore be an even more significant factor for survival of co-infected patients. Hospitalized pediatric patients with influenza and SARS-CoV-2 co-infections also had significantly lower rates of influenza vaccination (17%) relative to those with influenza alone (42%, p = 0.02) (Adams et al., 2022). These national data therefore complement our findings of a high degree of viral exclusion between SARS-CoV-2 and influenza, and suggest that the relatively few patients who develop co-infection may represent a particularly susceptible segment of the pediatric population, with reduced immunity to influenza.

Most studies have found comparably low rates of viral co-infection in adult populations as those we present here. For example, Chen and Er (2022) characterized FilmArray™ RP results from 804 Emergency Department patients in Taiwan, with 27.9% having positive results, 5.3% of patients having co-infection, but only two co-infections involving SARS-CoV-2, with the plurality of co-infections involving Adenovirus and Rhino/Enterovirus (42%) (Chen and Er, 2022). However, these authors did little to examine the rates of specific viral co-infections relative to the frequency each virus was encountered, or to explore correlations between the incidence of specific viral co-infections. A recent metanalysis of viral co-infections in the setting of COVID-19 found a similar rate of co-infection (5.0%) (Krumbein et al., 2023), but the most prevalent co-infecting virus was influenza (1.5%), followed by enterovirus (1.3%), and co-infections were more common in children (9.4%) than adults (3.5%). However, this meta-analysis was conducted on studies published from late 2019 to August 2021, and therefore did not include the most recent two respiratory viral seasons (Krumbein et al., 2023). Similarly, rates of respiratory viral co-infection among COVID-19 patients have been consistently higher in children, with one study reporting up to 15.8% for hospitalized patients, and 33.9% among children less than 5 years of age (Wanga et al., 2021), consistent with the findings in our study.

One limitation of the present study is the inability to distinguish the timeline of multiple infections, with each RPP datapoint representing a snapshot in time. Therefore differentiating superinfection vs. early co-infection was not possible. This makes it more challenging to determine which viruses from co-infected patients increased or decreased the likelihood of infection with another virus, or whether other factors, such as ineffective clearance of a virus, may have been responsible for affinity interactions seen. We suspect that this latter scenario played a role in the overall increased rate of co-infection we saw for adenovirus, and the relatively low exclusion or increased affinity of adenovirus for other viruses. Schjelderup Nilsen et al. (2019) found higher positivity rates of adenovirus in healthy children relative to those with respiratory tract infection, though symptomatic children were significantly more likely to have only adenovirus DNA detected (mono-infection), grow adenovirus in culture and have higher adenovirus viral loads (Schjelderup Nilsen et al., 2019). These authors therefore concluded that qualitative PCR testing for adenovirus DNA alone was not useful in the pediatric population as a diagnostic test. Zadheidar et al. (2022) found similar rates of adenovirus positivity in both symptomatic children and healthy controls, but found different subtypes predominated in each population (Zadheidar et al., 2022). The increased rates of co-infection involving adenovirus seen here may therefore be partially attributable to asymptomatic colonization.

We also cannot distinguish from our analysis whether reduced probability of viral co-infection was due to, or contributed to, distinct trends in monthly incidence in nearly each viral category assessed over the study period. Due to the limited number of co-infections for some targets, such as individual parainfluenza viruses, coronaviruses, and influenza viruses, their incidence was combined by group, which prevents assessment for specific viruses within each category, such as some of those observed by Horemheb-Rubio et al. (2022). For example, these authors found increases in the probability of interaction between specific HPIV 4 and influenza A(H3N2), as well as HPIV 1 and HCOV-NL63, while we found overall decrease in the interaction probability of these viral categories, but were not able to assess affinity between specific viruses within each category (Horemheb-Rubio et al., 2022). Additionally, viral exclusion/affinity analysis was not performed for individual months in the dataset due to the relatively infrequent co-infections for a given month. However, when we focused on November 2022, a month in which SARS-CoV-2, influenza and RSV were all present at relatively high incidence, we found rates of co-infection of each viral pair comparably low as to results obtained for the full 14 months assessed by this study (**Figure S1**). Thus, viral co-infection of these viruses is present at rates far lower than would be expected during a month where each is present at high incidence, suggesting that some biological mechanism of viral exclusion or host response also plays a significant role. This may have reduced the sensitivity of our analysis of viral co-infection due to the non-linear relationship between the probability of co-infection and the monthly prevalence of each virus involved

In contrast to the present study, affinity of adenovirus towards co-infection with seasonal coronaviruses was not observed by Horemheb-Rubio et al. Whereas the previous study observed a weak exclusionary interaction between adenovirus and RSV, rhinovirus/enterovirus, and parainfluenza, this interaction was not seen in the present study. However, a weak viral exclusion between adenovirus and both influenza and RSV was consistently observed in both studies (Horemheb-Rubio et al., 2022). The differences between the two studies could have stemmed from a variety of factors in addition to the methodological differences noted above, including differences in the pediatric host population, changes in viral interactions, viral immunity occurring in the setting of SARS-CoV-2, or other viral interactions that cannot be determined here. Further, the reduced incidence of overall viral infection, and particularly viral co-infection, seen for the adult population, did not allow for a robust comparison in the adult population. However, greatly reduced rates of all respiratory viral co-infections, even relative to overall infections in adults, suggests that viral exclusion may be even more common among adults. Though differences in RPP ordering practices make it likely that some proportion of this difference is from artifact due to the different clinical thresholds for which the test was used in these populations.

In summary, the data presented here demonstrate that, despite the expectedly high rates of RPP positivity seen in the pediatric population, viral co-infection occurred significantly less frequently than would be predicted from viral incidence alone. However, viruses included in the panel displayed a range of predilections for co-infection, with Adenovirus and non-SARS-CoV2 Coronaviruses demonstrating the highest frequency of co-infection, while SARS-CoV2 and influenza demonstrated the lowest overall. The distinct peaks in positivity rate for each virus over the course of the study period suggest that low co-infection rates may be in part due to differences in their distribution over time, but biological exclusion of viruses present in the same population likely also play a significant role. Further study is necessary to distinguish to what extent low rates of viral co-infection seen here can be attributed to different temporal trends of viral incidence, biological mechanisms of viral exclusion or virally-induced changes in host immune defense.

## Data availability statement

The data analyzed in this study is subject to the following licenses/restrictions: Data was extracted from our laboratory information system and has sensitive patient information. Requests to access these datasets should be directed to FW, fw108@cumc.columbia.edu for access to de-identified data from preliminary analysis.

## Ethics statement

The studies involving human participants were reviewed and approved by Columbia University Institutional Review Board. Written informed consent from the participants’ legal guardian/next of kin was not required to participate in this study in accordance with the national legislation and the institutional requirements.

## Author contributions

FW and MW designed the study and submitted for expedited IRB review. FW extracted the data from the LIS and MW performed the data analysis, with feedback from FW. MW composed the manuscript and figures, with initial review by FW. DG and GB revised the manuscript and figures prior to submission. All authors contributed to the article and approved the submitted version.

## Supplementary material

Supplementary Figure 1 Specific viral co-infection incidence in November, 2022. **(A)** The percentage of RPPs positive for each viral target, or group of targets (influenza), relative to total RPPs ordered for November, 2022. **(B)** The expected incidence (as percentage of total RPPs) of each type of co-infection based on the individual viral incidences listed in **(A)**, relative to the actual incidence of each viral co-infection (as percentage of total RPPs ordered) for November, 2022. **(C)** The percent decrease from the expected incidence of each viral co-infection to the actual incidence reported in **(B)**.
