# Supplementary figures and images for: Assessing respiratory viral exclusion and affinity interactions through co-infection incidence in a pediatric population during the 2022 resurgence of influenza and RSV
Source: Front Cell Infect Microbiol. 2023 Jun 14;13:1208235. doi: 10.3389/fcimb.2023.1208235 (PMC10302716; doi:10.3389/fcimb.2023.1208235)

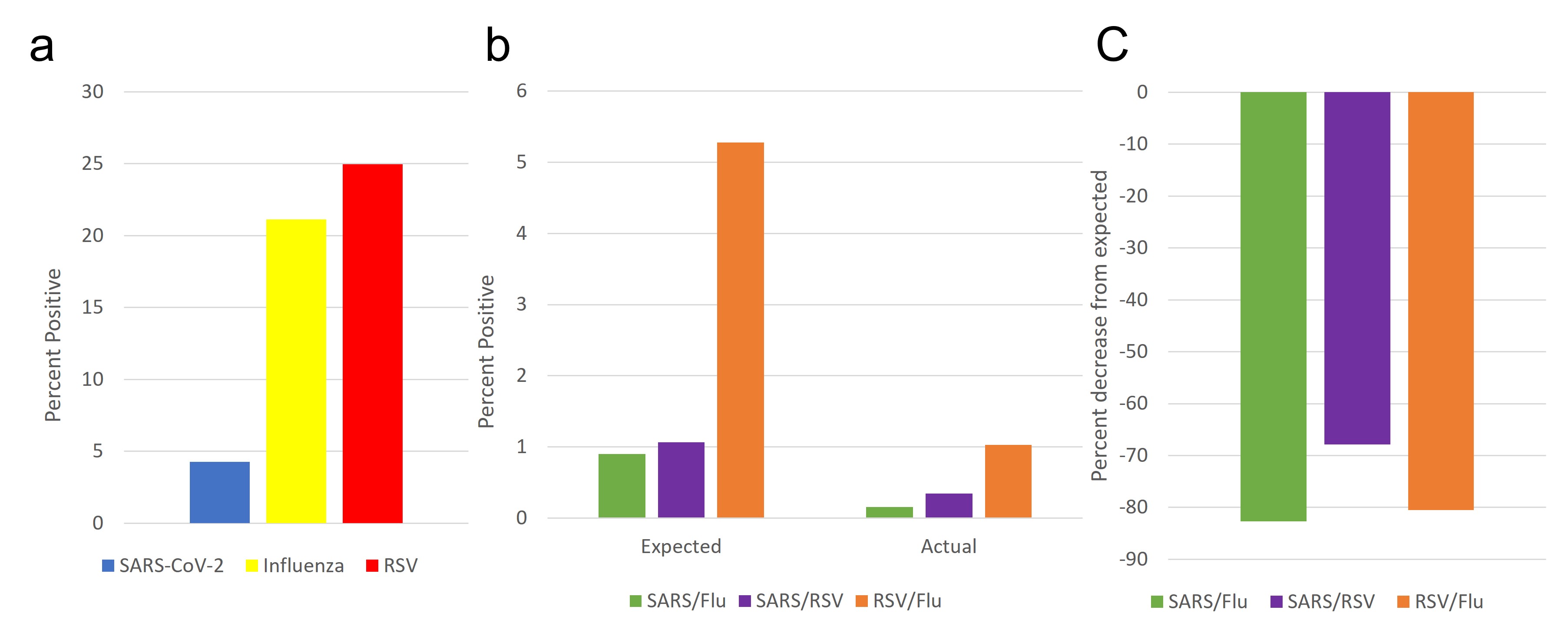

Supplement: Supplementary Figure 1 — Specific viral co-infection incidence in November, 2022. (A) The percentage of RPPs positive for each viral target, or group of targets (influenza), relative to total RPPs ordered for November, 2022. (B) The expected incidence (as percentage of total RPPs) of each type of co-infection based on the individual viral incidences listed in (A), relative to the actual incidence of each viral co-infection (as percentage of total RPPs ordered) for November, 2022. (C) The percent decrease from the expected incidence of each viral co-infection to the actual incidence reported in (B). [file Image_1.jpeg]
